# Inter- and Intra-subtype genotypic differences that differentiate *Mycobacterium avium* subspecies *paratuberculosis* strains

**DOI:** 10.1186/1471-2180-12-264

**Published:** 2012-11-19

**Authors:** Franck Biet, Iker A Sevilla, Thierry Cochard, Louise H Lefrançois, Joseba M Garrido, Ian Heron, Ramón A Juste, Joyce McLuckie, Virginie C Thibault, Philip Supply, Desmond M Collins, Marcel A Behr, Karen Stevenson

**Affiliations:** 1 INRA, UMR1282, Infectiologie Santé Publique (ISP-311), Nouzilly, F-37380, France; 2 Neiker-tecnalia, Dpto. de Producción y Sanidad Animal, Berreaga 1, Derio, Bizkaia, 48160, Spain; 3 Moredun Research Institute, Pentlands Science Park, Bush Loan, Penicuik, Scotland, EH26 0PZ, UK; 4 INSERM, Lille, U1019, France; 5 CNRS UMR, Lille, 8204, France; 6 Institut Pasteur de Lille, Center for Infection and Immunity of Lille, Lille, France; 7 Univ Lille Nord de France, Lille, France; 8 AgResearch, Wallaceville, Upper Hutt, P.O. Box 40063, New Zealand; 9 McGill University, Montreal, Quebec, H3G 1A4, Canada

## Abstract

**Background:**

*Mycobacterium avium* subspecies *paratuberculosis* (Map) is the aetiological agent of Johne’s disease or paratuberculosis and is included within the *Mycobacterium avium* complex (MAC). Map strains are of two major types often referred to as ‘Sheep’ or ‘S-type’ and ‘Cattle’ or ‘C-type’. With the advent of more discriminatory typing techniques it has been possible to further classify the S-type strains into two groups referred to as Type I and Type III. This study was undertaken to genotype a large panel of S-type small ruminant isolates from different hosts and geographical origins and to compare them with a large panel of well documented C-type isolates to assess the genetic diversity of these strain types. Methods used included Mycobacterial Interspersed Repetitive Units - Variable-Number Tandem Repeat analysis (MIRU-VNTR), analysis of Large Sequence Polymorphisms by PCR (LSP analysis), Single Nucleotide Polymorphism (SNP) analysis of *gyr* genes, Pulsed-Field Gel Electrophoresis (PFGE) and Restriction Fragment Length Polymorphism analysis coupled with hybridization to IS*900* (IS*900*-RFLP) analysis.

**Results:**

The presence of LSP^A^4 and absence of LSP^A^20 was confirmed in all 24 Map S-type strains analysed. SNPs within the gyr genes divided the S-type strains into types I and III. Twenty four PFGE multiplex profiles and eleven different IS*900*-RFLP profiles were identified among the S-type isolates, some of them not previously published. Both PFGE and IS*900*-RFLP segregated the S-type strains into types I and III and the results concurred with those of the *gyr* SNP analysis. Nine MIRU-VNTR genotypes were identified in these isolates. MIRU-VNTR analysis differentiated Map strains from other members of *Mycobacterium avium* Complex, and Map S-type from C-type but not type I from III. Pigmented Map isolates were found of type I or III.

**Conclusion:**

This is the largest panel of S-type strains investigated to date. The S-type strains could be further divided into two subtypes, I and III by some of the typing techniques (IS*900*-RFLP, PFGE and SNP analysis of the *gyr* genes). MIRU-VNTR did not divide the strains into the subtypes I and III but did detect genetic differences between isolates within each of the subtypes. Pigmentation is not exclusively associated with type I strains.

## Background

The aetiologic agent of Johne’s disease or paratuberculosis, *M. avium* subsp*. paratuberculosis* (Map), is one of the subspecies included in the *Mycobacterium avium* Complex (MAC). Based on the comparison of whole-genomes of Map*,* a biphasic evolution scheme has been proposed distinguishing two major lineages, a sheep lineage and a cattle lineage [1]. In addition to genotypic differences [2,3], strains belonging to these two lineages exhibit phenotypic differences including growth rate [2-4], utilization of different iron metabolic pathways [4], profile of cytokine responses induced in bovine macrophages [5] or transcriptional profiles in a human macrophage model [6]. The association of each lineage with either the sheep or cattle host is not exclusive since strains representative of either lineage can cause disease in all types of ruminants. Historically, strains belonging to the sheep lineage have been referred to as ‘Sheep or S-type’ and those of the cattle lineage ‘Cattle or C-type’ according to the species from which they were first isolated. As the technologies for molecular typing advanced and more genotyping studies were undertaken, greater genetic diversity was detected within both the S- and C-type strains. Pulsed-field gel electrophoresis (PFGE) revealed three strain types designated Types I, II and III [7,8]. Type II is synonymous with C-type and types I and III comprise the S-type. In this paper we will use the term S-type to describe collectively type I and III strains and have designated the types I and III as subtypes. S-type strains have not been characterized to the same extent as C-type strains due to the difficulty in culturing the strains *in vitro* resulting in a limited number of strains available for such studies. Here we undertook the first comprehensive genotyping study of a large representative panel of S-type strains using various typing methods that have been applied to Map strains, individually or in combinations, to draw a portrait of S-type strains. We studied both inter and intra-subtype genotypic strain differences using restriction fragment length polymorphism analysis coupled with hybridization to IS*900* (IS*900* RFLP), PFGE and various PCRs based on variable-number tandem repeat (VNTR) loci and mycobacterial interspersed repetitive units (MIRUs) [9,10] MIRU-VNTR typing [11], the presence or absence of large sequence polymorphisms (LSPs) [12] and the *gyr*A and B genes [13]. Our panel of S-type strains comprised strains from different geographic origins with different restriction enzyme profiles and includes pigmented strains. We also incorporated typing data obtained for additional Map C-type isolates to represent the all diversity of the genotypes described and *Mycobacterium. avium* subsp. *avium* (Maa) *Mycobacterium. avium* subsp. *silvaticum* (Mas) and *Mycobacterium avium* subsp. *hominissuis* (Mah) for comparison.

## Methods

### Panel of strains

A total of 24 Map S-type isolates were obtained from Scotland (n = 6), Spain (n = 11), Canada (n = 2), New Zealand (n = 2), Faroe Islands (n = 2) and Iceland (n = 1), isolated from sheep, goats and a pig (see Table 1 and Additional file 1: Table S1). Isolates were propagated on slopes of one of the following media depending on what was used routinely in the supply laboratories: modified Middlebrook 7H11 supplemented with 20% (vol/vol) heat-inactivated newborn calf serum, 2.5% (vol/vol) glycerol, 2 mM asparagine, 10% (vol/vol) Middlebrook oleic acid-albumin-dextrose-catalase (OADC) enrichment medium (Becton Dickinson, Oxford, Oxfordshire, United Kingdom), Selectatabs (code MS 24; MAST Laboratories Ltd., Merseyside, United Kingdom), and 2 μg ml^-1^ mycobactin J (Allied Monitor, Fayette, Mo.); Herrold’s egg yolk medium with 2 μg ml^−1^ mycobactin J or Lowenstein-Jensen medium with 2 μg ml^−1^ mycobactin J. In addition, genotyping information obtained previously [11,18,19] from 148 Map C-type strains, 31 Maa isolates, 4 Mas isolates and 82 Mah isolates were used for comparison and phylogenetic analyses and are described in Additional file 1: Table S1.

**Table 1:** Synthesis of information and genotyping data of S strains of Map by subtype

**Strain**	**Subtype**	**Origin**		**Profile**				**MIRU- VNTR Patterns**^**5**^	**SNP Gyr A & B**	**LSP**^**6**^**A20 4**		**Pigmentation**	**Strains References**
		**Host**	**Country**^**1**^** regions**	**RFLP**^**2**^	**PFGE**^**3**^	**MIRU- VNTR**^**4**^							
6756		Ovine	NZ	S1	nd	INMV	72	41331118	A/C C/C	-	+		[14]
6759		Ovine	NZ	S1	nd	INMV	72	41331118	A/C C/C	-	+		[14]
P133/79		Ovine	FO	S2	nd	INMV	70	71331118	A/C C/C	-	+		[15]
21P		Ovine	FO	S2	[9]	INMV	70	71331118	A/C C/C	-	+	+	[8]
235 G	I	Ovine	UK, Shetland	S2	[75–8]	INMV	70	71331118	A/C C/C	-	+	+	[8]
M189		Ovine	UK, Scotland	S2	[7]	INMV	21	51331118	A/C C/C	-	+	+	[8]
M15/04		Ovine	UK, Scotland	S2	[nd-70]	INMV	70	71331118	A/C C/C	-	+	+	[8]
M254/04		Ovine	UK, Scotland	S2	nd	INMV	32	61331118	A/C C/C	-	+	+	This study
M71/03		Ovine	UK, Scotland	S2	[80–71]	INMV	70	71331118	A/C C/C	-	+	+	This study
M72/03		Ovine	UK, Scotland	S2	[77–70]	INMV	70	71331118	A/C C/C	-	+	+	This study
22 G		Ovine	ES, Basque	A	[69–50]	INMV	84	91331118	A/T C/T	-	+		[16]
OVICAP16		Caprine	ES, Andalucia	A	[65–61]	INMV	85	(11)1331118	A/T C/T	-	+		[16]
OVICAP49		Ovine	ES, Navarra	A	[57–57]	INMV	70	71331118	A/T C/T	-	+	+	This study
21I		Ovine	ES, Basque	B	[61–47]	INMV	70	71331118	A/T C/T	-	+		[16]
PCR311		Caprine	ES, Balearic	B	[16–47]	INMV	70	71331118	A/T C/T	-	+		[16]
19I		Ovine	ES, Basque	C	[79–55]	INMV	70	71331118	A/T C/T	-	+		[16]
85/14	III	Ovine	CA	C	nd	INMV	27	81331118	A/T C/T	-	+		[14]
OVICAP34		Ovine	ES, Basque	D	[66–62]	INMV	21	51331118	A/T C/T	-	+		This study
18I		Ovine	ES, Basque	E	[67–51]	INMV	84	91331118	A/T C/T	-	+		[16]
FO21		Ovine	ES, Aragon	F	[56–56]	INMV	84	91331118	A/T C/T	-	+	+	This study
LN20		Porcine	CA	I1	nd	INMV	71	51131118	A/T C/T	-	+		[14]
269OV		Ovine	ES, Basque	I10	[69–54]	INMV	72	41331118	A/T C/T	-	+		[16]
M284/08		Ovine	ES, Basque	I10	[71–64]	INMV	72	41331118	A/T C/T	-	+		[16]
P465		Ovine	IS	I2	nd	INMV	73	20331118	A/T C/T	-	+		This study

^1^ Country and regions: ES, Spain; CA, Canada; UK, United Kingdom; FO, Faroe Island; IS, Iceland; NZ, New Zealand.

^2^ nd, not determined; alphanumeric nomenclature as defined by Pavlik *et al*., 1999 [17], alphabetic nomenclature correspond to new profiles identified in this study.

^3^ Nomenclature as defined by Stevenson *et al*., 2002 [8].

^4^ Nomenclature as defined by Thibault *et al*., 2007 [11].

^5^ Number of repeats at locus 292-X3-25-47-3-7-10-32 defined by Thibault *et al.,* 2007 [11].

^6^ +, presence; -, absence.

### IS*900*-RFLP method

Map strains were typed by IS*900*-RFLP as described previously [11]. Profiles were designated according to nomenclature previously described [17,20-22]. Profiles were analysed using Bionumerics™ software version 6.5 (Applied Maths, Belgium).

### PFGE analysis

PFGE analysis was carried out using *Sna*BI and *Spe*I according to the published standardized procedure of Stevenson *et al*. [8] with the following modifications. Plugs were prepared to yield a density of 1.2 × 10^10^ cells ml^-1^ and the incubation time in lysis buffer was increased to 48 hr. The concentration of lysozyme was increased to 4 mg ml^-1^. Incubation with proteinase K was carried out for a total of seven days and the enzyme was refreshed after four days. Restriction of plug DNA by *Spe*I was performed with 10U overnight after which the enzyme was refreshed and incubated for a further 6 hr. The parameters for electrophoresis of *Spe*I restriction fragments were changed to separate fragments of between 20 and 250Kb as determined by the CHEF MAPPER and electrophoresis was performed for 40 hr. Gel images were captured using an Alphaimager 2200 (Alpha Innotech). Profiles were analysed using Bionumerics™ software version 6.5 (Applied Maths, Belgium).

### SNP analysis of *gyr*A and *gyr*B genes

Primers (Additional file 2: Table S2) were designed for both *gyr*A (GenBank accession no. 2720426 [Genome number: NC_002944.2]) and *gyr*B genes (GenBank accession no. 2717659 [Genome number: NC_002944.2]). The PCR mixture was composed as follows using the GoTaq Flexi DNA polymerase (Promega). Two microliters of DNA solution was added to a final volume of 50 μl containing 0.2 μl of GoTaq Flexi DNA polymerase (5 U/μl), 2 mM (each) dATP, dCTP, dGTP, and dTTP (Promega); 10 μl of 5x PCR buffer supplied by the manufacturer; 1 μM of each primers; and 1.5 mM of MgCl_2_. The reactions were carried out using a TC-512 thermal cycler (Techne). PCR conditions were as follows: 1 cycle of 5 min at 94°C; 30 cycles of 30 s at 94°C, 30 s at 58°C, and 30 s at 72°C; and 1 cycle of 7 min at 72°C. PCR products were visualized by electrophoresis using 1.5% agarose gels (agarose electrophoresis grade; Invitrogen), purified using NucleoSpin® Extract II (Macherey-Nagel) and sequenced by GenomExpress (Grenoble, France). Sequence analysis and SNP detection were performed by using the Bionumerics™ software version 6.5 (Applied Maths, Belgium).

### LSP analysis

Primers were used according to Semret *et al*. [12] and described in Additional file 2: Table S2. The PCR mixture comprised 2 μl of DNA solution added to a final volume of 50 μl containing 0.2 μl of GoTaq Flexi DNA polymerase (5 U/μl Promega), 2 mM (each) dATP, dCTP, dGTP, and dTTP (Promega); 10 μl of 5x PCR buffer supplied by the manufacturer; 1 μM of each primers; 1 μL of dimethyl sulfoxide (Sigma) and 1.5 mM of MgCl_2_. The reactions were carried out using a TC-512 thermal cycler (Techne). PCR conditions were as follows: 1 cycle of 5 min at 94°C; 30 cycles of 30 s at 94°C, 30 s at 55°C, and 30 s at 72°C; and 1 cycle of 7 min at 72°C. To detect presence or absence of each LSP, PCR products were analyzed by electrophoresis using 1.5% agarose gels.

### MIRU-VNTR analysis

DNA in agarose plugs prepared for PFGE analysis was used for MIRU-VNTR analysis according to Stevenson et al. [23]. Small pieces of agarose plug, approximately 2 mm thick, were washed in TE buffer (pH 8) to remove residual EDTA in the storage buffer. One hundred microlitres of TE buffer were added to the agarose and the sample boiled for 10 min to melt the agarose. Five microlitres were used for PCR and MIRU-VNTR analysis interrogating eight polymorphic loci was performed as described by Thibault *et al.*[11]. The allelic diversity (*h*) at a locus was calculated by using Nei’s index (see Additional file 3: Table S4) *h* = 1 − ∑ *x*_*i*_^*2*^*n*/(*n* − 1)], where *x*_*i*_ is the frequency of the *i*^th^ allele at the locus, and *n* the number of isolates [24].

### Calculation of the discriminatory power

The Simpson Discrimination Index (DI) described by Hunter and Gaston [25] was used as a numerical index for the discriminatory power of each typing method PFGE, IS*900*-RFLP and MIRU-VNTR and combinations of the typing methods (see Table 2 and Additional file 3: Table S4). The DI was calculated using the following formula:

(1) $$DI=1-\left[\frac{1}{N\left(N-\right)}\sum_{j=1}^{s}n_{j}\left(n_{j}-1\right)\right]$$

**Table 2:** **Discriminatory Index of IS*900 *RFLP, MIRU-VNTR and *SnaB1, Spe1 *PFGE typing used alone and in combination**

**Typing methods**		**S type**			**C type**^**1**^	**All types**
	Subtypes	I	III	I + III	II	
IS*900* RFLP	No.^2^	10	14	24	35	59
	DI	0.356	0.934	0.873	0.644	0.856
PFGE *Sna*B1	No.	5	10	15	24	39
	DI	1.000	0.956	0.990	0.895	0.960
PFGE *Spe*1	No.	5	10	15	24	39
	DI	1.000	0.978	0.990	0.801	0.924
PFGE (*Sna*B1-*Spe*1)	No.	5	10	15	24	39
	DI	1.000	1.000	1.000	1.000	1.000
MIRU-VNTR	No.	10	14	24	35	59
	DI	0.644	0.89	0.801	0.876	0.925
IS*900* RFLP + MIRU-VNTR	No.	10	14	24	35	59
	DI	0.644	0.736	0.935	0.965	0.977

^1^: Panel of strains selected to represented the whole diversity of RFLP and MIRU-VNTR profiles of type C strains.

^2^: No. Number of strains analyzed.

Where *N* is the total number of isolates in the typing scheme, *s* is the total number of distinct patterns discriminated by each typing method and strategy, and *n*_*j*_ is the number of isolates belonging to the *j*th pattern.

## Results

### LSP analysis

**Figure 1:**
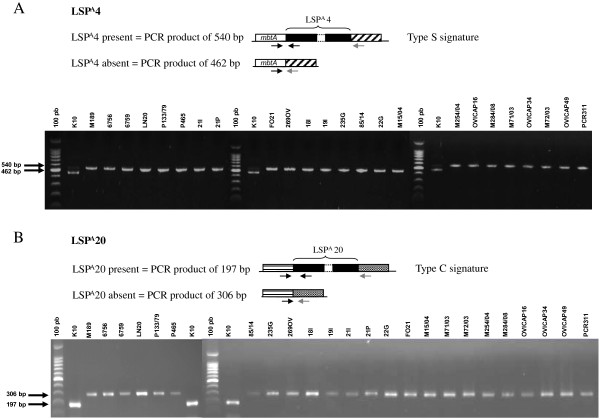
Detection of types and subtypes of strains based on of the absence or presence of large sequences LSPA4 (A) and LSPA20 (B) investigated by PCR.

The existence of two major Map lineages was previously supported by the distribution of two LSPs specific for Map as described by Semret *et al*. [12,26,27]: the region corresponding to LSP^A^20 is specifically absent from strains of the sheep lineage whereas that corresponding to LSP^A^4 is specifically absent from strains of the cattle lineage. The distribution of these LSPs was thus investigated across our representative panel of Map S-type strains from various origins. As shown in Figure 1, analysis by PCR supports the association of the LSP^A^20 region with C-type strains whereas the LSP^A^4 region is present in all S-type strains. Presence of the LSP^A^4 region was not related to PFGE subtype I versus III, of the country of origin and pigmentation status (Table 1).

### SNP analysis

Since SNPs found in *gyr*A and B genes have been reported to be subtype (I, II, III)-specific, the panel of Map S-type strains was subjected to SNP analysis and compared to C type K-10 strain. As shown in Table 3, consensus sequences obtained matched those previously published and distinguished types I, II and III of Map.

**Table 3:** **SNPs found in *gyrA *and *gyrB *genes for *M. avium *subsp. *paratuberculosis *strain K-10 and *M. avium *subsp. *paratuberculosis *types I and III**

**Strains**	**Type**	**IS*900* RFLP profiles**	***gyr*A**		***gyrB***	
			**position 1822**	**1986**	**1353**	**1626**
K10*	II	R01	CCCGAGGAGCGGATCGCT-	ACTCGTGGGCGCGGTGTTGT	CCGGTCGACCGATCCGCGC-	CCAGCACATCTCGACGCTGT
6756	I	S1	.....A....-	..........	......C...-	..........
6759	I	S1	.....A....-	..........	......C...-	..........
P133/79	I	S2	.....A....-	..........	......C...-	..........
21P	I	S2	.....A....-	..........	......C...-	..........
235 G	I	S2	.....A....-	..........	......C...-	..........
M189	I	S2	.....A....-	..........	......C...-	..........
M15/04	I	S2	.....A....-	..........	......C...-	..........
M254/04	I	S2	.....A....-	..........	......C...-	..........
M71/03	I	S2	.....A....-	..........	......C...-	..........
M72/03	I	S2	.....A....-	..........	......C...-	..........
22 G	III	A	.....A....-	.....T.....	......C...-	.....T.....
OVICAP16	III	A	.....A....-	.....T.....	......C...-	.....T.....
OVICAP49	III	A	.....A....-	.....T.....	......C...-	.....T.....
21I	III	B	.....A....-	.....T.....	......C...-	.....T.....
PCR311	III	B	.....A....-	.....T.....	......C...-	.....T.....
19I	III	C	.....A....-	.....T.....	......C...-	.....T.....
85/14	III	C	.....A....-	.....T.....	......C...-	.....T.....
OVICAP34	III	D	.....A....-	.....T.....	......C...-	.....T.....
18I	III	E	.....A....-	.....T.....	......C...-	.....T.....
FO21	III	F	.....A....-	.....T.....	......C...-	.....T.....
LN20	III	I1	.....A....-	.....T.....	......C...-	.....T.....
269OV	III	I10	.....A....-	.....T.....	......C...-	.....T.....
M284/08	III	I10	.....A....-	.....T.....	......C...-	.....T.....
P465	III	I2	.....A....-	.....T.....	......C...-	.....T.....
Consensus	I		CCCGAGGAGAGGATCGCT-	ACTCGTGGGCGCGGTGTTGT	CCGGTCGACCGACCCGCGC-	CCAGCACATCTCGACGCTGT
Consensus	III		CCCGAGGAGAGGATCGCT-	ACTCGTGGGTGCGGTGTTGT	CCGGTCGACCGACCCGCGC-	CCAGCACATTTCGACGCTGT
K10	II		CCCGAGGAGCGGATCGCT-	ACTCGTGGGCGCGGTGTTGT	CCGGTCGACCGATCCGCGC-	CCAGCACATCTCGACGCTGT

*K10 sequenced strain type II used as sequence reference.

### PFGE typing

**Figure 2:**
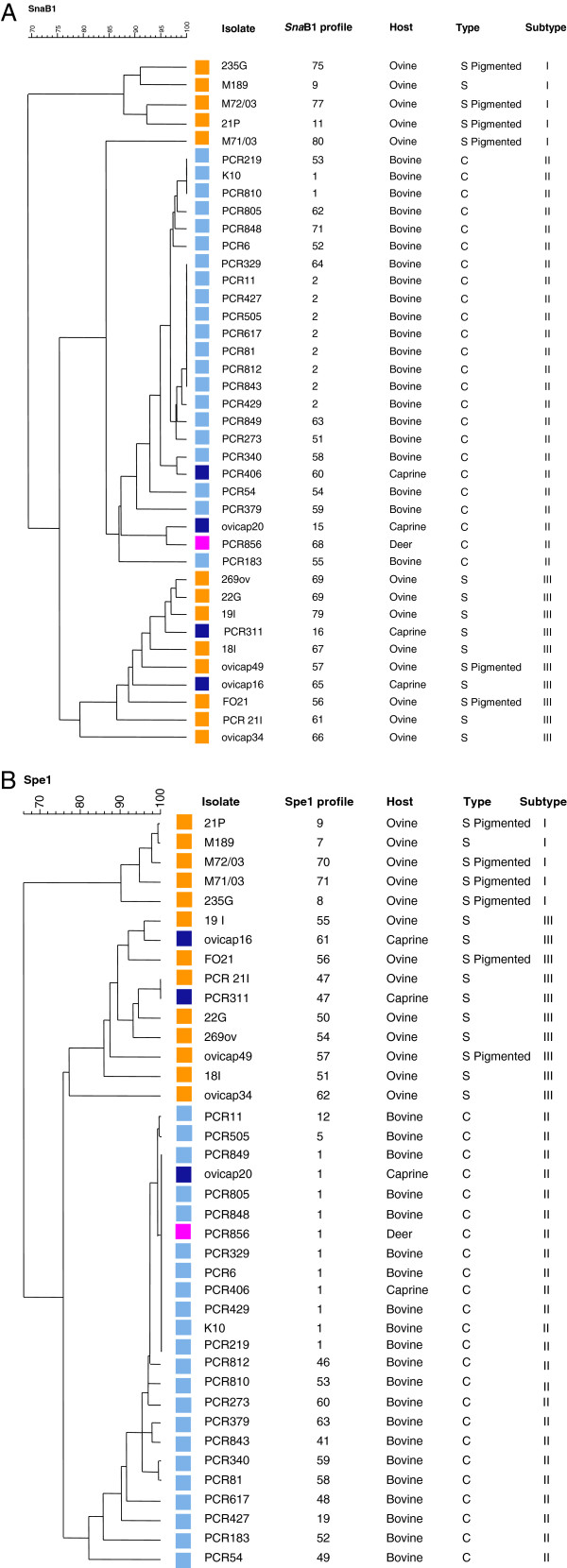
**UPGMA Dendrogram showing the profiles of Map strain obtained by PFGE using *Sna*B1 (A) or (B) *Spe*1.** The numbering codes of the profiles obtained for each enzyme were assigned according to the nomenclature available at http://www.moredun.org.uk/PFGE-mycobacteria. The colored squares indicate the animal origin of strains: cattle (sky blue), sheep (orange), goat (dark blue) and deer (purple).

PFGE analysis results were obtained for 15 S-type and 24 C-type strains (Figure 2A and 2B). The sequenced K10 type II strain was also included. *Sna*B1 or *Spe*I analyses segregated strains according to the two sheep and cattle lineages and at the subtype level I, II and III. With *Sna*BI and *Spe*I individually, 5 different profiles were obtained for the 5 type I strains and 9 different profiles for the 10 type III strains. The type II strains exhibited 15 different *Sna*BI profiles, with profile [2] being the most frequent (8 strains) and 14 different *Spe*I profiles with profile [1] being the most frequent (11 strains). The DI of the subtype I and subtype III were respectively 1 and 0.956 for *Sna*B1 and 1 and 0.978 for *Spe*I and that of C-type (Type II) was 0.895 for *Sna*BI and 0.801 for *Spe*I (see Table 2 and Additional file 3: Table S4). DI of 0.96 and 0.924 for *Sna*BI and *Spe*I respectively was achieved for the 39 Map strains presented in Figure 2A and 2B. The combination of both enzymes gave 39 unique multiplex profiles (see Table 1 and Additional file 1: Table S1).

### IS*900*-RFLP typing

**Figure 3:**
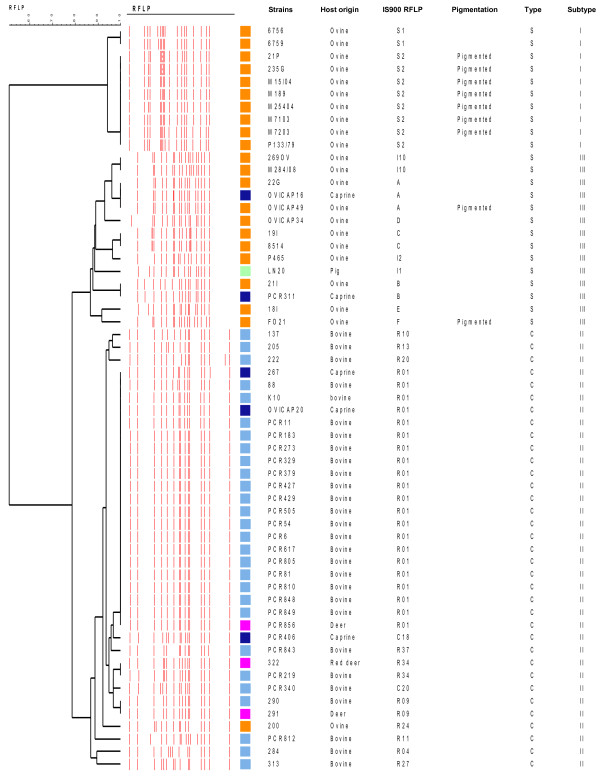
**UPGMA dendrogram based on IS*900 *RFLP typing, using *Bst *EII on a panel of strains of S-type and C-types.** 28 strains were isolated from cattle (sky blue), 22 from sheep (orange), 5 from goat (dark blue), 3 from deer (purple) and one from pig (light green).

IS*900*-RFLP typing clearly separated the strains into three groups that correlate with the PFGE subtypes I, II and III (Figure 3). Ten strains of S-type, subtype I cluster into two groups of profiles S1 (n = 2) and S2 (n = 8). The 14 strains of S-type, subtype III display more polymorphism with 9 profiles, including 6 new ones. Profiles previously described included I1 (n = 1), I2 (n = 1) and I10 (n = 2). The new profiles were called A (n = 3), B (n = 2), C (n = 2), D, E and F (n = 1 each) (indicated in the Additional file 4: Figure S1). The strains of C-type were well distinguished from S-type and were not highly polymorphic. In this panel of strains the most widely distributed profile R01 was found for 21 strains, then R09 (n = 2) and R34 (n = 2) and 10 profiles were identified in only one isolate, R04, R10, R11, R13, R20, R24, R27, R37, C18 and C20. With this Map panel of strains the discrimination index (DI) of RFLP was shown very variable depending on the type and the subtype of the strains. The DI of the subtype I was very low (0.356), for the subtype III high (0.934) and that of C-type (Type II) was low (0.644) (Table 2). A DI of 0.856 was achieved for the 59 Map strains presented in Figure 3.

### MIRU-VNTR typing

**Figure 4:**
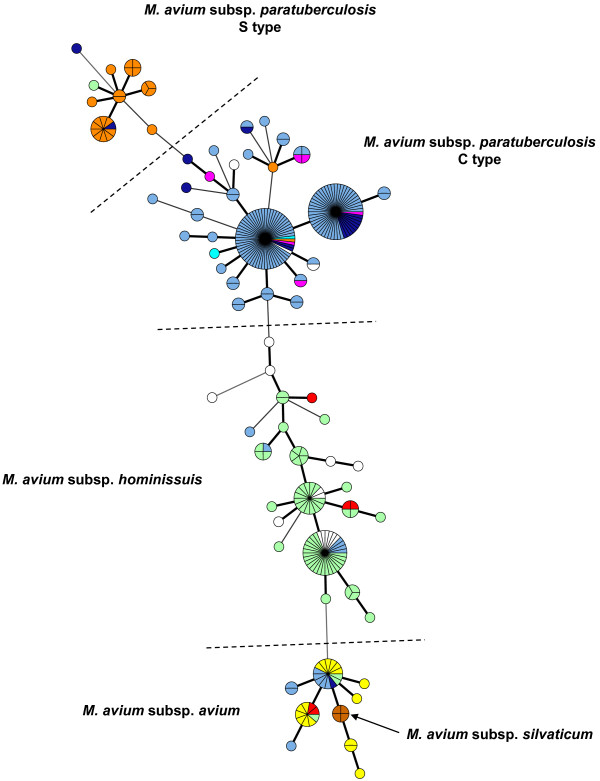
**Minimum spanning tree based on MIRU-VNTR genotypes among *Mycobacterium avium *subsp. ***paratuberculosis * of types S and C, *Mycobacterium avium* subsp. *avium*, *Mycobacterium avium* subsp. *hominissuis*, and *Mycobacterium avium* subsp. *silvaticum*. 135 strains were isolated from cattle (sky blue), 23 strains from sheep (orange), 17 strains from goat (dark blue), 63 strains from pigs (light green), 17 strains from birds (yellow), 17 strains from humans (white), 6 strains from deer (purple), 5 strains from other sources (red), 4 strains from wood pigeons (brown), and 2 different vaccine strains (316 F from France and United Kingdom) (light blue). Each genotype is displayed as a pie chart, the size of which is proportional to the number of strains, with color-coded distribution of the strain origins. The number of loci differing between the genotypes is indicated by the style of the connecting lines: thick and short, 1 difference; intermediate, 2 differences; thin and long: 3 differences.

The result of MIRU-VNTR typing of the S-type strains is shown in Table 1. MIRU-VNTR data from 148 C-type (type II) strains previously described [11,18,19] were included in the analysis (see Additional file 1: Table S1). MIRU-VNTR using the eight markers described previously [11] could differentiate between S- and C-type strains but not between the subtypes I and III. On this panel of strains, type III strains were the most polymorphic with a DI of 0.89 compared to 0.644 for type I strains and 0.876 for type II strains selected to represent the diversity of INMV profiles described. INMV profiles 21, 70 and 72 were shared by both type I and III strains. As described previously [11] IS*900* RFLP and MIRU-VNTR typing may be used in combination to gain higher resolution. This was verified also on this panel of strains including S-type. In total, the combination of the two methods distinguished 32 distinct patterns comprising 59 isolates. Therefore, using carefully on the same set of strains, a DI of 0.977 was achieved for this panel by using IS*900* RFLP and MIRU-VNTR typing in combination compared to 0.856 for IS*900* RFLP typing alone and 0.925 for MIRU-VNTR typing (Table 2 and Additional file 3: Table S4). Because MIRU-VNTR is applicable to all members of the MAC, we wanted to know how the INMV profiles segregated within the MAC. None of the INMV profiles identified in the S-type strains matched those of other MAC members. The results presented by the minimum spanning tree in Figure 4, show that Map S-type strains are clearly separated from Map C-type strains, including 113 strains previously typed, and also from any strains belonging to the other subspecies *hominissuis*, *avium* or *silvaticum*. The allelic diversities of the various loci are shown in Additional file 5: Table S3. Five markers were monomorphic in Map S subtype III and 7 in Map S subtype I. In terms of the discriminatory hierarchy, locus 292 displayed the highest allelic diversity for both S- and C-type strains. This study shows that genotyping with MIRU-VNTR can distinguish MAC isolates to the species level and also distinguish with MAP subspecies to the strain type level.

## Discussion

In comparison to Map C-type strains, investigation of the epidemiology and genetics of S-type strains has been hampered due to difficulties in their isolation and their extremely slow growth-rate in laboratory culture [28,29]. Indeed, the isolation and maintenance of Map S-type strains continues to be a challenge for laboratories worldwide and relative to Map C-type strains a paltry number are available for study. Nowadays representative genome sequences are available for both C- and S-type subtype III Map strains [30,31]. This has facilitated the identification of specific genetic elements that can be used to identify isolates and discriminate between types and, in some cases subtypes of strains [14,16,22,32-34]. In this study we assembled a panel of S-type strains from different geographic origins and host species and undertook extensive molecular typing to improve our knowledge on the genetic diversity of these strains and their phylogenetic relationship with respect to Map C-type strains and other members of MAC. This is the largest panel of S-type strains investigated to date. Additionally, the study also permitted identification of the most efficient typing techniques for S-type strains. The results of the study coupled with previous results on genotypic and phenotypic characterization of Map strains concur with the division of this subspecies into two major lineages comprising S-type and C-type strains. However, the results of IS*900*-RFLP, PFGE and SNP analysis of the *gyr* genes clearly divide Map strains into three subtypes, Type II or C strains, Type I and Type III strains. But from the data available on these strains, the two subtypes do not seem to be associated with a particular phenotype and may just reflect regional genetic differences. Type I was first proposed to describe a group of ovine pigmented Map strains with distinctive PFGE profiles [8]. However, as more ovine strains were typed by PFGE, it became apparent that there was another cluster of non-pigmented ovine Map strains that were designated Type III strains [7]. The pigmented phenotype consequently became associated with the Type I strains. However, in this study we included two pigmented strains originating from different geographic locations, which were typed as type III by SNP analysis of the *gyr* genes, IS*900* RFLP and PFGE. The pigmentation phenotype is not therefore restricted to type I and there is no other obvious phenotype currently known to differentiate between types I and III. MIRU-VNTR, despite being highly discriminatory between strains did not separate the S-type strains into the two types I and III. There is therefore an argument for simplifying the current nomenclature for Map strain grouping [14]. Due to the historical nomenclature, to the absence of other comprehensive studies including all strain types and typing methods, to the inability of several techniques to distinguish between Type I and III and to the genetic and phenotypic similarities found between them in previous studies, we propose that S- and C-type nomenclature could be used to denote the two major groups or lineages and the Type I and III used to distinguish subtypes within S-type strains as we have done in this paper. In agreement with previous studies both PFGE and IS*900*-RFLP revealed little heterogeneity between isolates of the S subtype I. By comparison, this study shows that strains of S subtype III are more polymorphic. Diverse genotypes clustered within S subtype III have been identified circulating in small regional areas in Spain or even in the same farm [34], making more evident the higher heterogeneity of these strains. Interestingly, as far as we know no evidence of S subtype I strains has been found in Spain, a country with a significant sample of S-type strains in our panel and in previous works [8,16].

For molecular epidemiology (i.e. strain tracking), of the typing techniques used MIRU-VNTR would be the preferred technique for studying S-type strains. This technique gave a high discriminatory index with the eight loci employed in this study and could segregate the different members of MAC and the Map S- and C-type strains, although it has limitations in that it cannot differentiate between the subtypes I and III. For detecting genetic variability between S-type strains the number of loci used could be reduced to 3 (292, X3 and 25). The greatest genetic variation occurred at locus 292 with S-type strains typically having a much higher number of repeats than C-type strains (up to 11 were detected in this study). No more than 4 repeats at locus 292 were detected in C-type strains. The locus 292 locus is flanked by loci MAP2920c and MAP2921c referenced as acetyltransferase and quinone oxidoreductase, respectively. There has been only one other report of MIRU-VNTR typing of S-type strains [22]. In the latter study MIRU-VNTR loci 3 and 7 were thought to be of special importance for identifying subtype III strains but only two subtype III strains were typed. In our study all 14 subtype III and 10 subtype I strains had the same, one-repeat unit alleles at each of these two loci, as found in the two strains typed previously [22]. Although uncommon, a few C-type strains in this study were also found with a single copy at these loci so this is even not unique to S-type strains. All Mah, Maa and Mas strains tested in this study also had one repeat unit at locus 3 and all Maa and 61% of Mah strains had a single copy at locus 7. The discriminatory power of MIRU-VNTR to differentiate between the subtypes I and III could be improved by identifying additional loci. Although MIRU-VNTR cannot distinguish between the subtypes I and III, currently it is the only PCR-based typing technique to reveal significant genetic diversity between S-type strains useful for epidemiological investigations. The technique requires only a small amount of DNA and can therefore be carried out on single colonies as well as cell pellets from liquid culture systems. LSP analysis rapidly differentiates the S-type from C-type strains by the absence of LSP^A^20 and presence of LSP^A^4 but provides no information regarding genetic diversity within S-type strains. SNP analysis of the *gyr* genes is more complex requiring sequencing of the PCR product to differentiate between S- and C-types and between subtypes I and III [13]. However, the S subtype information would be of limited value for epidemiological studies and tracing the source of infection. Furthermore, as we become better at isolating S-type strains and type more strains it is likely that further S subtypes will become apparent. PFGE and IS*900*-RFLP both give good discrimination between the Map strain types and subtypes but require larger amounts of high quality DNA, which necessitates *in vitro* growth of the strains and therefore is not ideal for S-type strains.

## Conclusions

This is the largest panel of S-type strains investigated to date. The S-type strains can be further divided into two types, I and III, by some (IS*900*-RFLP, PFGE and SNP analysis of the *gyr* genes) but not all (not by MIRU-VNTR typing) of the typing techniques. Pigmentation is not exclusively associated with S subtype I strains. Therefore, a simplified nomenclature is proposed designating types I and III as subtypes of S-type strains. The epidemiological and phylogenetic significance of S type subdivision into I and III subtypes needs, however, to be further clarified. Molecular typing using IS*900*-RFLP, PFGE and MIRU-VNTR demonstrates that S-type strains are genetically diverse, subtype III being the most heterogeneous group. Due to the scarcity of S-type strains in culture, typing techniques have been largely optimized using C-type strains. Further genomic sequencing of S-type strains should reveal variable genetic loci unique to S-type strains that could be exploited to further improve discrimination of S-type strains. Genome sequence data of isolates belonging to subtypes I and III should ultimately clarify the phylogeny and provide a framework to classify different phenotypic, pathogenic and epidemiological characteristics of Map strains.

## Competing interests

The authors have no competing interests.

## Authors’ contributions

FB, IS and KS conceived of the study, participated in its design and coordination, collated and analysed the data and drafted the manuscript. TC, LL, JG, IH, JM and VT participated in the laboratory and field work. RJ, TC, LL, PS participated in analysing the data. All authors read, criticized and approved the final manuscript.

## Supplementary Material

Additional file 1 **Table S1.** Description of the strains used in this study indicating their origins and details of their genotypes and phenotypes data. Click here for file

Additional file 2 **Table S2.** The table shows the primers sequences used in this study. Click here for file

Additional file 3 **Table S4.** The table details the calculation of the Discriminatory Index for each typing methods including IS*900* RFLP, MIRU-VNTR and PFGE (*Sna*B1, *Spe*1) used alone and in combination. The table details the calculation of the allelic diversity (*h*) at a locus MIRU-VNTR using Nei’s index. Click here for file

Additional file 4 **Figure S1.** The figure shows the new IS*900* RFLP profiles obtained from analysis with strains S of subtype III. Click here for file

Additional file 5 **Table S3.** The table describes the MIRU-VNTR allelic distribution among the strains of Map of type S and C and other Mac members. Click here for file
