# Supplementary figures and images for: Inter- and Intra-subtype genotypic differences that differentiate Mycobacterium avium subspecies paratuberculosis strains
Source: BMC Microbiol. 2012 Nov 19;12:264. doi: 10.1186/1471-2180-12-264 (PMC3546927; doi:10.1186/1471-2180-12-264)

## Slide 1
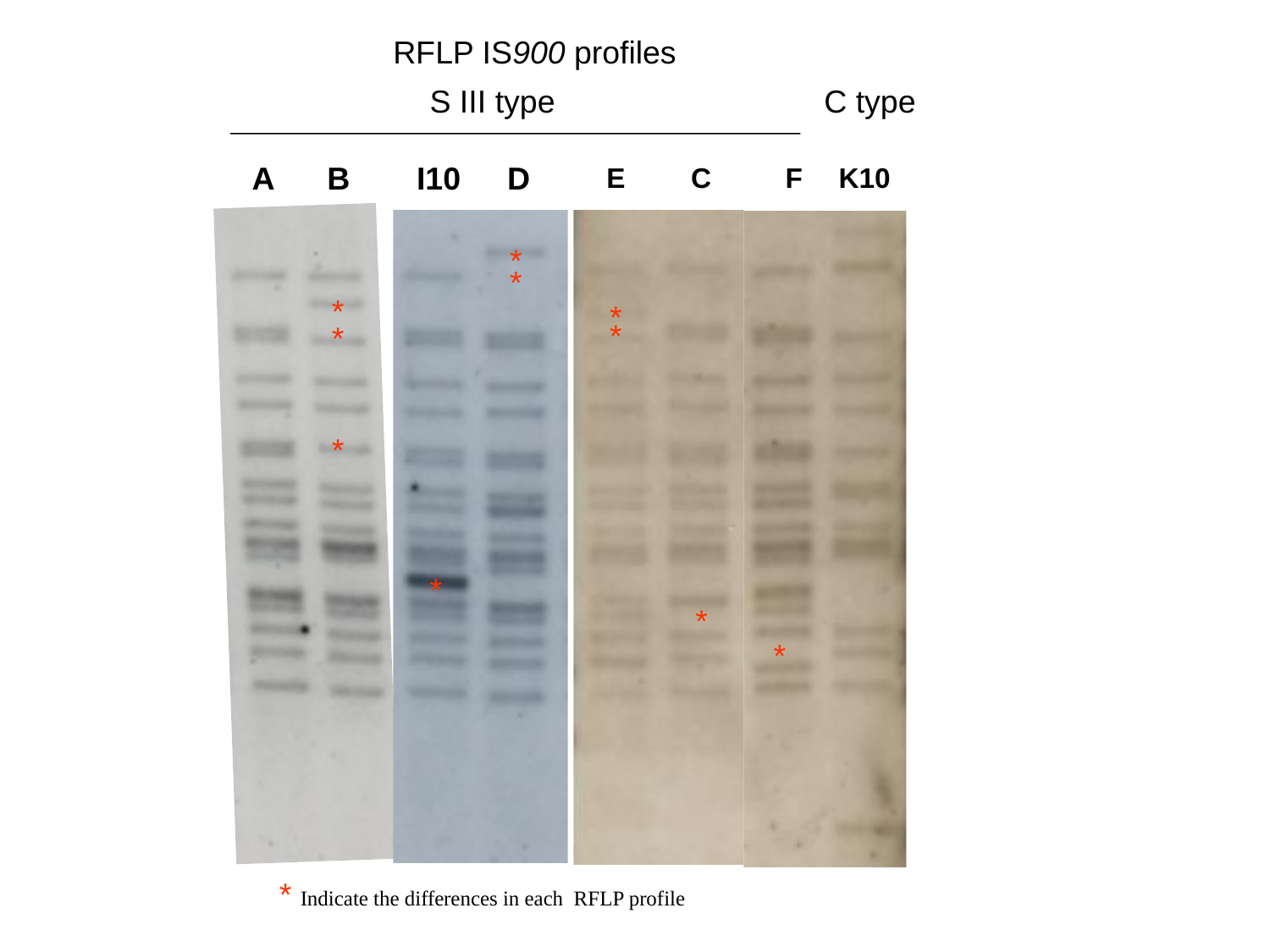

RFLP IS900 profiles
S III type
C type
A
B
I10
D
E
C
F
K10
*
*
*
*
*
*
*
*
*
*
* Indicate the differences in each RFLP profile

Supplement: Additional file 4 — Figure S1. The figure shows the new IS900 RFLP profiles obtained from analysis with strains S of subtype III. [file 1471-2180-12-264-S4.ppt]
