# Supplementary material for: Inter- and Intra-subtype genotypic differences that differentiate Mycobacterium avium subspecies paratuberculosis strains
Source: BMC Microbiol. 2012 Nov 19;12:264. doi: 10.1186/1471-2180-12-264 (PMC3546927; doi:10.1186/1471-2180-12-264)
Supplement: Additional file 5 — Table S3. The table describes the MIRU-VNTR allelic distribution among the strains of Map of type S and C and other Mac members. [file 1471-2180-12-264-S5.docx]

**Table S3**. MIRU-VNTR allelic distribution among Map of type S and C and other Mac member

| Strains  panel | locus | No. of isolates with the following MIRU-VNTR copy no.: | | | | | | | | | | | | Allelic  diversity (h)^a^ |
| --- | --- | --- | --- | --- | --- | --- | --- | --- | --- | --- | --- | --- | --- | --- |
|  |  | 0 | 1 | 2 | 3 | 4 | 5 | 6 | 7 | 8 | 9 | 10 | 11 |  |
|  | 292 |  |  | 1 |  | 4 | 3 | 1 | 10 | 1 | 3 |  | 1 | 0.750 |
|  | X3 | 1 | 23 |  |  |  |  |  |  |  |  |  |  | 0.040 |
| *Map* | 25 |  | 1 |  | 23 |  |  |  |  |  |  |  |  | 0.040 |
| S type | 47 |  |  |  | 24 |  |  |  |  |  |  |  |  | 0 |
|  | 3 |  | 24 |  |  |  |  |  |  |  |  |  |  | 0 |
|  | 7 |  | 24 |  |  |  |  |  |  |  |  |  |  | 0 |
|  | 10 |  | 24 |  |  |  |  |  |  |  |  |  |  | 0 |
|  | 32 |  |  |  |  |  |  |  |  | 24 |  |  |  | 0 |
|  |  |  |  |  |  |  |  |  |  |  |  |  |  |  |
|  | 292 |  |  |  |  | 2 | 1 | 1 | 6 |  |  |  |  | 0.533 |
|  | X3 |  | 10 |  |  |  |  |  |  |  |  |  |  | 0 |
| *Map* | 25 |  |  |  | 10 |  |  |  |  |  |  |  |  | 0 |
| S type | 47 |  |  |  | 10 |  |  |  |  |  |  |  |  | 0 |
| Subtype I | 3 |  | 10 |  |  |  |  |  |  |  |  |  |  | 0 |
|  | 7 |  | 10 |  |  |  |  |  |  |  |  |  |  | 0 |
|  | 10 |  | 10 |  |  |  |  |  |  |  |  |  |  | 0 |
|  | 32 |  |  |  |  |  |  |  |  | 10 |  |  |  | 0 |
|  |  |  |  |  |  |  |  |  |  |  |  |  |  |  |
|  | 292 |  |  | 1 |  | 2 | 2 |  | 4 | 1 | 3 |  | 1 | 0.802 |
|  | X3 | 1 | 13 |  |  |  |  |  |  |  |  |  |  | 0.066 |
| *Map* | 25 |  | 1 |  | 13 |  |  |  |  |  |  |  |  | 0.066 |
| S type | 47 |  |  |  | 14 |  |  |  |  |  |  |  |  | 0 |
| Subtype | 3 |  | 14 |  |  |  |  |  |  |  |  |  |  | 0 |
| III | 7 |  | 14 |  |  |  |  |  |  |  |  |  |  | 0 |
|  | 10 |  | 14 |  |  |  |  |  |  |  |  |  |  | 0 |
|  | 32 |  |  |  |  |  |  |  |  | 14 |  |  |  | 0 |
|  |  |  |  |  |  |  |  |  |  |  |  |  |  |  |
|  | 292 |  |  | 9 | 81 | 58 |  |  |  |  |  |  |  | 0.540 |
|  | X3 |  | 2 | 146 |  |  |  |  |  |  |  |  |  | 0.020 |
| *Map* | 25 |  |  |  | 139 | 1 | 8 |  |  |  |  |  |  | 0.109 |
| C type | 47 |  |  | 5 | 142 | 1 |  |  |  |  |  |  |  | 0.072 |
|  | 3 |  | 1 | 147 |  |  |  |  |  |  |  |  |  | 0.007 |
|  | 7 |  | 6 | 136 | 2 | 2 | 1 |  |  | 1 |  |  |  | 0.148 |
|  | 10 |  | 10 | 138 |  |  |  |  |  |  |  |  |  | 0.120 |
|  | 32 |  |  |  |  |  |  | 1 |  | 145 | 2 |  |  | 0.033 |
|  |  |  |  |  |  |  |  |  |  |  |  |  |  |  |
|  | 292 | 6 | 1 | 73 | 2 |  |  |  |  |  |  |  |  | 0.192 |
|  | X3 |  |  | 23 | 6 | 36 | 16 |  | 1 |  |  |  |  | 0.681 |
| *Mah* | 25 |  |  | 69 | 12 | 1 |  |  |  |  |  |  |  | 0.261 |
|  | 47 |  |  | 72 | 10 |  |  |  |  |  |  |  |  | 0.204 |
|  | 3 |  | 82 |  |  |  |  |  |  |  |  |  |  | 0 |
|  | 7 |  | 50 | 32 |  |  |  |  |  |  |  |  |  | 0.469 |
|  | 10 |  | 1 | 76 | 1 |  | 4 |  |  |  |  |  |  | 0.128 |
|  | 32 |  |  |  |  |  | 4 |  | 1 | 74 | 3 |  |  | 0.172 |
|  |  |  |  |  |  |  |  |  |  |  |  |  |  |  |
|  | 292 |  |  | 31 |  |  |  |  |  |  |  |  |  | 0 |
|  | X3 |  | 3 | 2 | 10 | 15 | 1 |  |  |  |  |  |  | 0.635 |
| *Maa* | 25 |  | 30 | 1 |  |  |  |  |  |  |  |  |  | 0.031 |
|  | 47 |  |  | 1 | 30 |  |  |  |  |  |  |  |  | 0.031 |
|  | 3 |  | 31 |  |  |  |  |  |  |  |  |  |  | 0 |
|  | 7 |  | 31 |  |  |  |  |  |  |  |  |  |  | 0 |
|  | 10 |  | 3 | 27 | 1 |  |  |  |  |  |  |  |  | 0.205 |
|  | 32 |  |  |  |  |  |  |  | 30 | 1 |  |  |  | 0.031 |

*^a^* Calculated by using Nei’s index. h. genetic diversity.
